# Supplementary figures and images for: Circadian clock proteins KaiB and Rbp2 of Synechococcus elongatus display oscillations in their subcellular localization patterns
Source: Microbiol Spectr. 2025 Dec 26;14(2):e01845-25. doi: 10.1128/spectrum.01845-25 (PMC12889050; doi:10.1128/spectrum.01845-25)

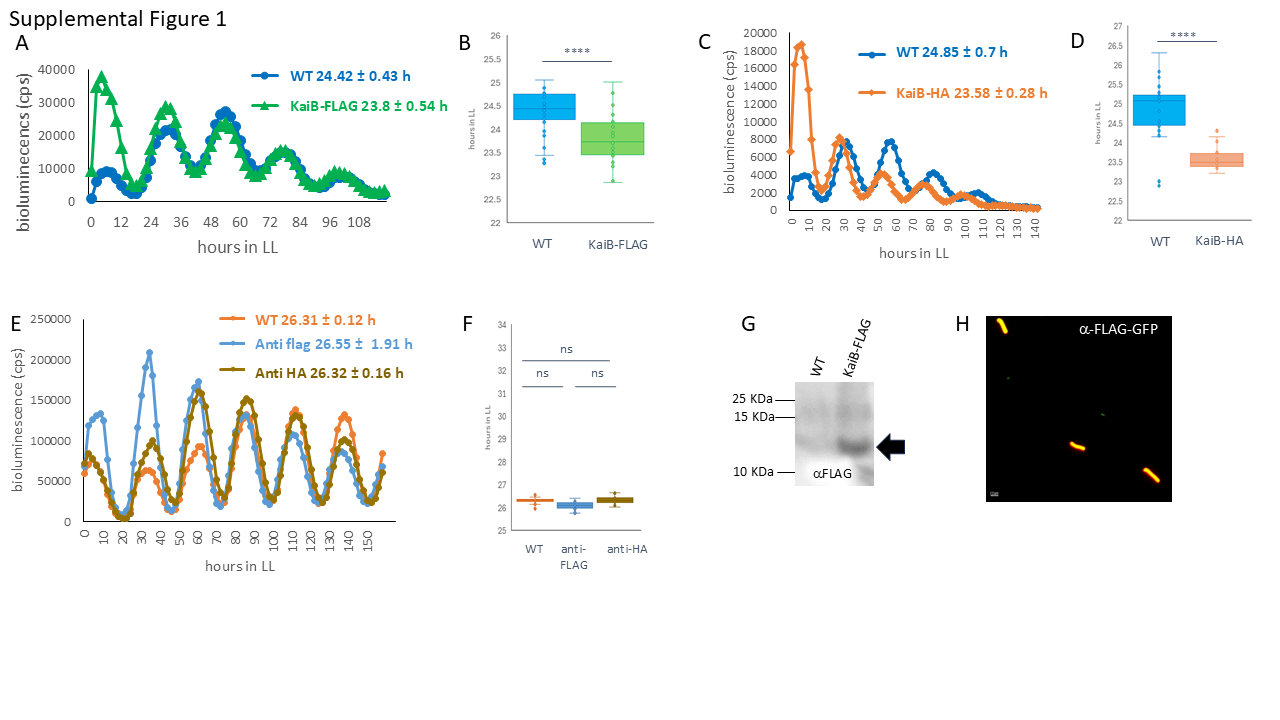

Supplement: Fig. S1 — Validation of strains used to determine KaiB subcellular localization. [file spectrum.01845-25-s0001.tif]

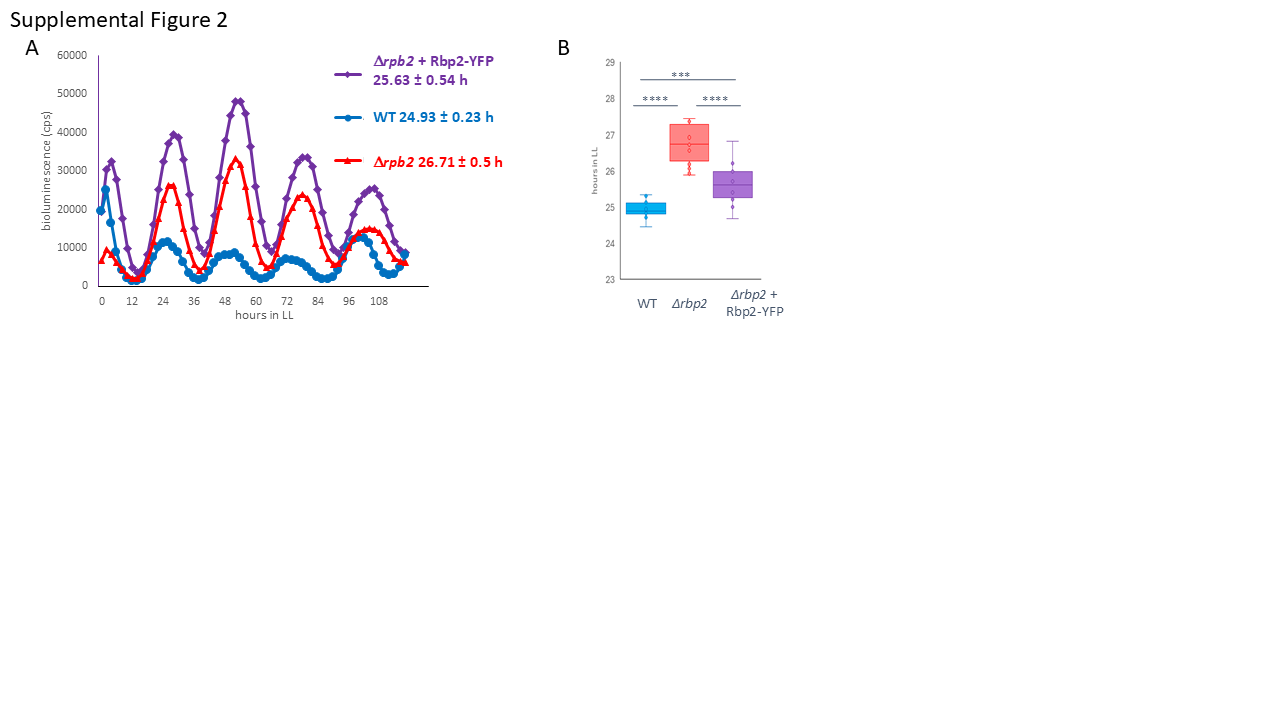

Supplement: Fig. S2 — Validation of strains used to determine Rbp2 subcellular localization. [file spectrum.01845-25-s0002.tif]
