# Supplementary material for: Circadian clock proteins KaiB and Rbp2 of Synechococcus elongatus display oscillations in their subcellular localization patterns
Source: Microbiol Spectr. 2025 Dec 26;14(2):e01845-25. doi: 10.1128/spectrum.01845-25 (PMC12889050; doi:10.1128/spectrum.01845-25)
Supplement: Supplemental material — Supplemental figure legends. [file spectrum.01845-25-s0003.docx]

**SUPPLEMENTARY FIGURE LEGENDS**

**Supplementary Figure 1. Validation of strains used to determine KaiB subcellular localization.** A) Bioluminescence monitoring of strains carrying a P*_kaiB_*-*luc* reporter. Expression of *kaiB*-FLAG from NS2 (green) has a period of 23.8 ± 0.54 h, similar to the wild type control (blue), which has a period of 24.4 ± 0.43h, demonstrating that C-terminally FLAG tagged variants of *kaiB* are functional. Wild type strain, blue, is AMC2133 (1), and expresses untagged *kaiB* from NS2. B) Bar graph showing statistical analysis of the periods reported in A). Error bars = standard deviation. ****p<0.00002, One-way ANOVA with Tukey’s post-hoc analysis. C) Bioluminescence monitoring of strains carrying a P*_kaiB_*-*luc* reporter. Expression of *kaiB*-HA from NS2 (orange), period of 23.58 ± 0.28 h, compared to the wild type control (blue), period of 24.85 ± 0.25 h. Wild type strain, blue, is AMC2133 (1), and expresses untagged *kaiB* from NS2. D) Bar graph showing statistical analysis of the periods reported in C). Error bars = standard deviation. ****p<0.00002, One-way ANOVA with Tukey’s post-hoc analysis. E) Bioluminescence monitoring of strain carrying a P*_kaiB_*-*luc* reporter shows that expression of the α-FLAG-GFP or α-HA-GFP frankenbodies does not affect the circadian clock. Strains in which the expression of the α-FLAG-GFP (blue) or α-HA-GFP (brown) frankenbodies was induced with theophylline, have a similar period the WT strain in blue. WT strain is *S. elongatus* transformed with pAM2226, expressing P*_kaiB_*-*luc* from NS1. Experiment was conducted in the presence of 2 mM theophylline in DMSO to induce the expression of α-FLAG-GFP frankenbody. F) Bar graph showing statistical analysis of the periods reported in E). Error bars = standard deviation. ns= not significant, One-way ANOVA with Tukey’s post-hoc analysis. G) Immunoblot using an antibody against the FLAG epitope shows that KaiB-FLAG is expressed as a full-length fusion protein. Arrow points to KaiB which has a molecular weight of 11.4 KDa. H) Representative fluorescent micrographs of cells expressing α-FLAG-GFP frankenbody induced with 2 mM theophylline in DMSO at ZT21, when KaiB localization is at it’s peak. The lack of foci observed demonstrates that the frankenbody alone does not form foci at night. Autofluorescence is shown in red. Scale bar = 2.5μm.

**Supplementary Figure 2. Validation of strains used to determine Rbp2 subcellular localization. A)** Bioluminescence monitoring of strain carrying a P*_kaiB_-luc* reporter shows that expression of Rbp2-YFP in a Δ*rbp2* mutant background (purple) has a period of 25.63 ±0.54 hr, which is more similar to the Δ*rbp2* mutant strain (red), 26.71 ± 0.5 hr, suggesting that the C-terminal fusion is not functional. WT strain is AMC2036 (blue), has a period of 24.93 ± 0.23 hr. B) Bar graph showing statistical analysis of the periods reported in A). Error bars = standard deviation. ****p<0.00002, One-way ANOVA with Tukey’s post-hoc analysis.

**SUPPLEMENTARY TABLES**

**Supplementary Table 1. Plasmids used in this study**

| Plasmid | Description | Source |
| --- | --- | --- |
| pLA0003 | C-terminal P*trc*-YFP-Rbp2, SpSm, expressed from NS1 | This work |
| pLA0006 | N-terminal P*trc*-YFP-Rbp2, SpSm, expressed from NS1 | This work |
| pLA0067 | C-terminal P*_kaiB_*-1xFLAG-KaiB, Nt, expressed from NS1 | This work |
| pLA0065 | C-terminal P*_kaiB_*-1xHA-KaiB, Nt, expressed from NS1 | This work |
| pAM5082 | C-terminal P*_kaiB_*-ECFP-KaiC, Gm, expressed from NS2 | This work |
| pLA0070 | P*_conII_*-RiboB-αFLAG-GFP-frankenbody, Gm, expressed from NS3 | This work |
| pLA0069 | P*_conII_*-RiboB-αHA-GFP-frankenbody, Gm, expressed from NS3 | This work |
| pAM2595 | P*_trc_*-*kaiC*, Km, expressed from NS2 | Lab stock |
| pAM4663 | P*_kaiB_*-*luc*, Cm, expressed from NS3 | (2) |
| pAM2226 | P*_kaiB_*-*luc*, SpSm, expressed from NS1 | Lab stock |
| pLA106 | P*_trc_*-YFP-Rbp2^R42AF44AF46A^, SpSm, expressed from NS1 | This work |

**SUPPLEMENTARY REFERENCES**

1. Chang YG, Cohen SE, Phong C, Myers WK, Kim YI, Tseng R, Lin J, Zhang L, Boyd JS, Lee Y, Kang S, Lee D, Li S, Britt RD, Rust MJ, Golden SS, LiWang A. 2015. A protein fold switch joins the circadian oscillator to clock output in cyanobacteria. Science 349:324-8.

2. Cohen SE, Erb ML, Selimkhanov J, Dong G, Hasty J, Pogliano J, Golden SS. 2014. Dynamic localization of the cyanobacterial circadian clock proteins. Curr Biol 24:1836-44.
